# MDR-1 and MRP2 Gene Polymorphisms in Mexican Epileptic Pediatric Patients with Complex Partial Seizures

**DOI:** 10.3389/fneur.2014.00184

**Published:** 2014-10-09

**Authors:** David Escalante-Santiago, Iris Angélica Feria-Romero, Rosa María Ribas-Aparicio, Dario Rayo-Mares, Pietro Fagiolino, Marta Vázquez, Consuelo Escamilla-Núñez, Israel Grijalva-Otero, Miguel Angel López-García, Sandra Orozco-Suárez

**Affiliations:** ^1^Programa de Biomedicina y Biotecnología Molecular, Escuela Nacional de Ciencias Biológicas del Instituto Politécnico Nacional, Mexico City, Mexico; ^2^Unidad de Investigación Médica en Enfermedades Neurológicas, Hospital de Especialidades, Centro Médico Nacional Siglo XXI, Instituto Mexicano del Seguro Social, Mexico City, Mexico; ^3^Neurología, Hospital de Pediatría, Centro Médico Nacional Siglo XXI, Instituto Mexicano del Seguro Social, Mexico City, Mexico; ^4^Departamento de Ciencias Farmacéuticas de la Facultad de Química, Universidad de la República, Montevideo, Uruguay; ^5^Instituto Nacional de Salude Publica, Cuernavaca, Mexico

**Keywords:** drug-resistant, epilepsy, *ABCB1*, *ABCC2*, Mexican patients, single-nucleotide polymorphisms, anti-epileptic drugs

## Abstract

Although the Pgp efflux transport protein is overexpressed in resected tissue of patients with epilepsy, the presence of polymorphisms in MDR1/*ABCB1* and MRP2/*ABCC2* in patients with antiepileptic-drugs resistant epilepsy (ADR) is controversial. The aim of this study was to perform an exploratory study to identify nucleotide changes and search new and reported mutations in patients with ADR and patients with good response (CTR) to antiepileptic drugs (AEDs) in a rigorously selected population. We analyzed 22 samples In Material and Methods, from drug-resistant patients with epilepsy and 7 samples from patients with good response to AEDs. Genomic DNA was obtained from leukocytes. Eleven exons in both genes were genotyped. The concentration of drugs in saliva and plasma was determined. The concentration of valproic acid in saliva was lower in ADR than in CRT. In *ABCB1*, five reported SNPs and five unreported nucleotide changes were identified; rs2229109 (GA) and rs2032582 (AT and AG) were found only in the ADR. Of six SNPs associated with the *ABCC2* that were found in the study population, rs3740066 (TT) and 66744T > A (TG) were found only in the ADR. The strongest risk factor in the *ABCB1* gene was identified as the TA genotype of rs2032582, whereas for the *ABCC2* gene the strongest risk factor was the T allele of rs3740066. The screening of SNPs in *ACBC1* and *ABCC2* indicates that the Mexican patients with epilepsy in this study display frequently reported *ABCC1* polymorphisms; however, in the study subjects with a higher risk factor for drug resistance, new nucleotide changes were found in the *ABCC2* gene. Thus, the population of Mexican patients with AED-resistant epilepsy (ADR) used in this study exhibits genetic variability with respect to those reported in other study populations; however, it is necessary to explore this polymorphism in a larger population of patients with ADR.

## Introduction

Epilepsy is a chronic non-communicable disorder of the brain that affects approximately 70 million people worldwide (1, 2). The administration of anti-epileptic drugs (AEDs) is the treatment of choice. In 30% of patients with epilepsy, seizures persist despite polytherapy with more than one AED, adequate monitoring of serum drug levels and the watchful care of a neurologist (3). Hence, the timely identification of patients who do not respond favorably to AEDs is crucial because these patients are affected both by the treatment and by continuing seizures. Although the pathophysiological basis of drug resistance in epilepsy is not clear, some mechanisms for this resistance have been proposed. Following seizures, the overexpression of drug-transporter proteins in the blood brain barrier (BBB) could be mediated by the nuclear receptor PXR (4); alternatively, polymorphisms in these proteins could abrogate their function. The main function of carrier proteins is to allow the passage of compounds through biological barriers such as the BBB. Using ATP, the ABC transporter family of proteins actively transports a wide variety of compounds, including toxins and xenobiotics, across cell membranes. ABC transporter proteins have two functional domains. The first domain is anchored to the cell membrane by its alpha-helical structure; the second, which is a nucleotide-binding domain (NBD), binds ATP, which provides the energy necessary for the conformational change that result in transport of the compound across the membrane. All ABC transporters have at least two transmembrane domains and two NBDs that are highly conserved between species (5).

MDR-1 (P-glycoprotein or P-gp) is an ATP-dependent efflux pump protein that controls the flow of toxins and drugs such as felbamate (FBM), gabapentin (GBP), lamotrigine (LTG), phenytoin (PTH), and topiramate (TPM) into the brain (6–9). The gene encoding MDR-1 is *ABCB1*, which is located on chromosome 7q21.12 and consists of 209,461 base pairs (bp) and 29 exons (NCBI reference sequence NG_011513.1). The most extensively studied single-nucleotide polymorphisms (SNPs) in the *ABCB1* gene are rs1128503 (**C**1236**T**), rs2032582 (**G**2677**T/A**), and rs1045642 (**C**3435**T**); the latter has received increased attention as a critical variable in resistance to AEDs (10–16) MRP2 is another efflux transporter protein present in the BBB. Its gene, *ABCC2*, is located on chromosome 10q24 and contains 69,000 bp representing 33 exons (NG_011798.1). The MRP2 transporter protein recognizes CBZ, LTG, and FBM; its most relevant polymorphisms are rs2273697 (G1249A) and rs3740066 (C3972T). The latter polymorphism reduces the transport of carbamazepine (17–20). The aim of this study was to perform a non-inferential exploratory study to identify reported nucleotide changes and explore new ones in a rigorously selected Mexican population of patients with AED-resistant epilepsy (ADR) and patients with good response to AEDs (CTR).

## Materials and Methods

### Patients and sample collection

This project was approved by the National Research Committee of the Instituto Mexicano del Seguro Social (Mexican Social Security Institute, IMSS). An observational study with 22 patients with ADR (cases) and 7 patients with good response to AEDs (controls) was performed, while avoiding inbreeding between the patient’s biological parents.

The inclusion criteria for ADR were patients with demonstrable epileptic focus through EEG and radiological focal structural lesion to discard and classified as resistant to pharmacological treatment, treated with two or more drugs (Table 1) at appropriate doses, serum levels within therapeutic range for at least 6 months of continuous treatment, and under the supervision of a neurologist pediatrician, with a frequency of three seizures per month, who attend Neurology Service, Hospital of Pediatrics, National Medical Center, Century XXI, who is of Mexican origin like their parents and grandparents, from 1 to 16 years of age, both genders, whereas for asymptomatic control patients was seizure-free for at least 6 months before the study.

**Table 1:** **Demographics and clinical data of patients with AEDs-resistant epilepsy and patients with good response to AEDs**.

Clinical data	ADR	CTR
Age (years ± SD), range	10.45 ± 3.762 (1–15)	11.50 ± 3.017 (1–15)
Gender (F/M)	12/8	3/3
Onset of seizures	4.715 ± 3.835 (1–14)	4.833 ± 3.656 (2–10)
Number of seizures/month	63.45 ± 27.76 (3–450)	–
Years of seizures	5.85 ± 0.8438 (1–12)	–
Controlled time (years ± SD) range	–	1.9 ± 0.28 (0.6–2)
**Valproic Ac. dose (mg/day)**	598.0 ± 129.7	814.3 ± 212.1
Valproic Ac. concentration/saliva (mg/l)	0.57 ± 0.12	1.68 ± 0.59
Valproic Ac. concentration/plasma (mg/l)	31.62 ± 8.03	35.17 ± 18.04
**Carbamacepine dose (mg/day)**	900.0 ± 100.0	600.0 ± 200.0
Carbamacepine concentration/saliva (mg/l)	0.7244 ± 0.3232	1.017 ± 0.034
Carbamacepine concentration/plasma (mg/l)	5.900 ± 0.156	6.76 ± 0.29
**Phenytoin dose (mg/day)**	145.0 ± 55.00	
Phenytoin concentration/saliva (mg/l)	0.9337 ± 0.06814	
Phenytoin concentration/plasma (mg/l)	3.150 ± 1.750	
**Levetiracetam dose (mg/day)**	1250 ± 322.7	1000 ± 0.0
Levetiracetam concentration/saliva (mg/l)	1.661 ± 0.8825	
Levetiracetam concentration/plasma (mg/l)	4.657 ± 0.5897	

*Mean ± SD*.

*F, female; M, male*.

Both groups had complex partial seizures, showed similar clinical variables, were previously selected under medical supervision, and gave written informed consent. Exclusion criteria were all types of seizures that are not complex partial, little adherence to treatment.

A total of 15 ml of blood was collected from each patient via antecubital vein using an EDTA tube, and two fractions of 0.5–2.0 ml of saliva were collected upon stimulation with citric acid. Samples that were used to monitor drug concentrations were taken before the first dose in the morning.

### Amplification and sequencing

Genomic DNA was extracted from leukocytes in patient blood samples using a commercial kit (Genomic DNA Purification kit, Thermo Scientific^®^, USA), according to the supplier’s recommendations. The polymorphisms (SNPs) from exons 1 (rs9282564), 10 (rs2229109), 11 (rs1128503), 13 (rs28381902), 20 (rs2032582), and 25 (rs1045642) were located in GenBank accession CCDS5608.1, corresponding to the *ABCB1* gene, and exons 10 (rs2273697), 25 (rs8187692, rs17222723), 28 (rs3740066), 29 (rs7899457), and 32 (rs8187710) were located in GenBank accession CCDS7484.1, corresponding to the *ABCC2* gene. These polymorphisms were selected based on their frequency in the Hispanic or Mexican population, as noted in NCBI GenBank. Each amplification reaction was performed with 5 μl (5 ng/μl) of genomic DNA in a 50 μl total reaction volume containing 5 μl of 10× reaction buffer, MgCl_2_ (concentration depending on the primer set), 1 μl 10 mMdNTPs (Thermo Scientific^®^, USA), 1 μl of each 10 mM flanking primer (Invitrogen^®^, USA), and 1 μl of Taq polymerase (Thermo Scientific^®^, USA). PCR was performed in a DNA engine system^®^thermocycler (Bio-Rad^®^, USA) with a cycle program at 94°C for 3 min, 38 cycles of 94°C for 30 s, $T_{h}^{\circ }$ for 35 s, 72°C for 35 s, and one extension cycle of 10 min at 72°C. The primers, MgCI_2_ concentration, and $T_{h}^{\circ }$ for each amplification reaction are listed in Table 2.

**Table 2:** **Primers used on ABCB1 and ABCC2 fragments amplification**.

GEN	EXON	Primer		MgCl_2_ (mM)	$T_{h}^{o}$	Size (bp)
		Fw	RV	
ABCB1	2	5′-AGGTTAGTCTCACCTCCAGCG-3′	5′-GGCTAGCTTGCGTTTCTTAAA-3′	1.0	57	270
	11	5′-ATTCGAAGAGTGGGCACAAA-3′	5′-TCATCTCACCATCCCCTCTGT-3′	1.0	52	398
	12	5′-ATTCGAAGAGTGGGCACAAA-3′	5′-TCATCTCACCATCCCCTCTGT-3′	1.0	52	398
	14	5′-TTGGGCTGTGTATAGGATTCC-3′	5′-AAGCCTCACTGACCTTATCCA-3′	1.5	55	274
	21	5′-CAGCATTCTGAAGTCATGGAA-3′	5′-TCCAAGAACTGGCTTTGCTA-3′	1.75	56	576
	26	5′-TGTGCTGGTCCTGAAGTTGAT-3′	5′-TGGTCGAACACTTTCATCCCT-3′	1.50	62	472
ABCC2	10	5′-TTAGGCATTGACCCTATCCA-3′	5′-GCCCAAACTCCCATTAAGAA-3′	1.25	56	366
	25	5′-CGGGACTGGCTGATTCTTTA-3′	5′-ATGGGTAAATACCCAGGGGAA-3′	1.0	54	273
	28	5′-TTCTATGACACGAGTCCTGGG-3′	5′-CATCCAGGCCTTCCTTCACT-3′	1.25	56	258
	29	5′-CCCCAAGAATTATTTGTGGAA-3′	5′-GCATGTGCCCGAGTAAGTT-3′	1.5	57	238
	32	5′-GCCTAGACTTGAGATGCTGC-3′	5′-TGGGGCCTTCTGCTAGAATT-3′	1.5	55	198

*The primer design was performed using the web page http://www.yeastgenome.org/cgi-bin/web-primer, $T_{h}^{o}$ were selected by a temperature gradient, and MgCl_2_ concentration was selected initially with 2.0 mM and performed gradually until obtained a single sharp band*.

The amplification products were purified with the GeneJET Gel Extraction kit (Thermo Scientific^®^, USA) and sequenced in a 5 μl total reaction volume using 100 ng of amplicon, Fw or Rv primer, and Big Dye 3.1 sequencing Ready Reaction Kit (Applied Biosystems, USA) according to the manufacturer’s recommendations. Sequencing was performed in a DNA engine system^®^thermocycler (Bio-Rad^®^, USA) with a cycle program at 94°C for 1 min, 30 cycles of 94°C for 10 s, 50°C for 5 s, 60°C for 4 min, and finally the fragments were analyzed in Automated Sequencing ABI Prism 310 Gene Analyzer (Applied Biosystems, USA), previous purification with DyeEx 2.0 Spin Kit (Qiagen, Germany).

### Drug monitoring

Plasma samples were used for VPA and CBZ detection by immunofluorescence with a polarized light system (FPIA, Dade Behring, USA), according to the supplier’s recommendations.

Saliva samples were used to detection concentration of CBZ and PHT (determined by Axsym^®^, Abbot Laboratories^®^, USA) according to the supplier’s recommendations, VPA, and LEV [determined by high performance liquid chromatography (HPLC)]. For VPA, 30 μl of an internal standard solution (98% octanoic acid diluted 1/500 with methanol), 200 μl of phosphoric acid, and 5 ml of cyclohexane were added to 1 ml of saliva and vortexed for 2 min. After centrifugation, a back-extraction was performed by adding 100 μl of 1% TEA to the organic phase supernatant. The mixture was vortexed and centrifuged. Finally, 20 μl of the aqueous phase was directly injected into a Dionex Ultimate 3000 Series. A Phenomenex Luna 5 μm CN column (15 cm × 4.6 mm) was used as the stationary phase. The mobile phase was a mixture of 40 mM phosphate potassium, monobasic, pH 3.4: Acetonitrile (90:10). The column compartment was kept at 40°C, and the wavelength of detection was 210 nm. For LEV, 50 μl of internal standard solution (diethylbarbituric acid, 12 mg/l) was added to 500 μl of saliva. For the extraction, 1.5 ml of ethyl acetate was added, and the mixture was vortexed for 1 min. After centrifugation, the supernatant was separated and dried under nitrogen stream at 37–40°C. Dry residue was dissolved with 100 μl of mobile phase, and 20 μl was injected into a Shimadzu LC-6A.A Phenomenex^®^ Luna C18 column (5 μm, 100 Å, 150 mm × 4.6 mm), which was used as a reversed stationary phase. The mobile phase was a mixture of 50 mM phosphate buffer and acetonitrile (72:8).

### Statistical analysis

Clinical data from patients were analyzed using non-parametric and unpaired tests (*p* < 0.05); allelic and genotype relative frequencies were analyzed using the Fisher exact test in Graph Pad Prism software version 4.00 (GraphpadPrisma Software Inc.^®^ San Diego, CA, USA). Hardy–Weinberg equilibrium (HWE) values were derived from chi-square tests, and the association between odds ratios (ORs) and confidence intervals was determined using software available at http://ihg.gsf.de/cgi-bin/hw/hwa1.pl.

## Results

### Clinical data

Because previous studies suggest that external factors such as gender, age of onset, number of seizures, and other factors influence the response to AED treatment, the selection of a homogeneous population for the study was an important aspect of this work (21, 22). There was no significant difference in male: female frequency between the two groups used in the study. Neither the average age of the patients (10.57 ± 1.56 for patients with good response to AEDs and 10.23 ± 0.78 for patients with ADR; *p* = 0.42) or the average age of onset of seizures (4.57 ± 1.43 years in patients with good response to AEDs and 4.38 ± 0.81 patients with ADR; *p* = 0.34) differed significantly. Furthermore, the average seizure-free time in patients with good response to AEDs was 1.9 years, while the average time with seizures in patients with ADR was 5.8 years with a frequency of 63.4 seizures per month. The AEDs VPA, CBZ, LTG, PHT, and LEV were used with relative frequencies of 0.71, 0.14, 0.29, 0.0, and 0.14, respectively, in patients with good response to AEDs and with relative frequencies of 0.59, 0.09, 0.0, 0.18, and 0.23, respectively, in patients with ADR. The most commonly used AED was VPA, but VPA and CBZ were prescribed to both groups (Table 1). The daily dose of VPA (814.3 ± 212.1 mg/day in patients with good response to AEDs and 598.0 ± 129.7 mg/day in patients with ADR; *p* = 0.206) was similar in the two groups. The two groups showed no significant difference in mean plasma VPA concentrations (35.17 ± 18.04 mg/l patients with good response to AEDs and 31.62 ± 8.03 mg/l in patients with ADR; *p* = 0.42); however, the average saliva drug concentration was lower in patients with ADR (1.68 ± 0.59 mg/l patients with good response to AEDs and 0.57 ± 0.12 mg/l in patients with ADR; *p* = 0.01).

### Polymorphisms in the *ABCB1* and *ABCC2* genes in Hispanic and Mexican populations

In this study, chromatograms from amplicons were analyzed to identify expected polymorphisms and to determine the relative frequencies of alleles and genotypes, as described in Table 3. For *ABCB1*, only the polymorphisms rs2229109, rs1128503, rs28381902, rs2032582, and rs1045642, but not rs9282564, were detected, whereas rs3740066 but not rs2273697, rs8187692, rs17222723, rs7899457, or rs8187710 were found in *ABCC2* in our patients, unlike the Hispanic/Mexican populations initially selected by the NCBI GenBank. In addition to these known polymorphisms, we identified rs2214102, rs2235047, and rs2235048 of *ABCB1* by sequencing the entire fragment including the exon and parts of the introns.

**Table 3:** **Genotype and allelic relative frequencies of SNPs, MAF value, amino acid changes, and OR found in *ABCB1* and *ABCC2* genes**.

Gene	SNP	Sample	Allele frequency	Genotype frequency	MAF	Protein association		Odds ratio		
						aa, change	Localization	Allele	Genotype	
									Heterozogous	Homozygous
*ABCB1*	rs2214102	CTR	G = 1.000	G/G = 1.000	A = 0.040/88	5′UTR region		**[G] *↔* [A], 2.446**	[GG] ↔ [GA], 2.692	[GG] ↔ [AA], 0.385
		ADR	G = 0.971	G/G = 0.955	
			A = 0.029	G/A = 0.045	
	rs2229109	CTR	G = 1.000	G/G = 1.000	A = 0.017/36	400; G > A; Ser > Asn	Cytoplasmatic; binding site; Second domain	[**G]** ***↔*** **[A], 1.706**	[GG] ↔ [GA], 1.829	[GG] ↔ [AA], 0.366
		ADR	G = 0.955	G/G = 0.909	
			A = 0.045	G/A = 0.091	
	rs1128503	CTR	T = 0.571	C/T = 0.571	T = 0.422/919	412; T > C; Gly > Gly	Cytoplasmatic; beta chain; second domain	**[T]** ***↔*** **[C], 1.543**	[TT] ↔ [TC], 1.200	[TT] ↔ [CC], 3.000
			C = 0.429	T/T = 0.286	
				C/C = 0.143	
		ADR	T = 0.455	C/T = 0.545	
			C = 0.545	T/T = 0.182	
				C/C = 0.273	
	rs2032582	CTR	G = 0.500	G/T = 0.429	A = 0.340/741	893; T > A; Ser > Thr	Cytoplasmatic; Alfa-helix chain; third domain	**[T]** ***↔*** **[A], 2.143**	[TT] ↔ [TA], 2.778	[TT] ↔ [AA], 0.526
			T = 0.500	G/G = 0.286		893; T > G; Ser > Ala
				T/T = 0.286				**[T]** ***↔*** **[G], 1.375**	[TT] ↔ [TG], 1.333	[TT] ↔ [GG], 1.750
		ADR	G = 0.523	G/T = 0.364	
			T = 0.409	G/G = 0.318	
			A = 0.068	T/T = 0.182	
				A/G = 0.045	
				A/T = 0.091	
	rs1045642	CTR	T = 0.429	TT = 0.143	T = 0.397/864	1145; T > C Ile > Ile	Cytoplasmatic, fourth domain	[T] ↔ [C], 0.825	[TT] ↔ [TC], 0.500	[TT] ↔ [CC], 0.600
			C = 0.571	TC = 0.571	
				CC = 0.286	
		ADR	T = 0.475	TT = 0.255	
			C = 0.525	TC = 0.450	
				CC = 0.300	
	rs2235047	CTR	T = 0.857	TT = 0.714	G = 0.179/389	Intron between 25 and 26 exon		**[T]** ***↔*** **[G], 1.020**	[TT] ↔ [TG], 1.026	[TT] ↔ [GG], 0.407
			G = 0.143	TG = 0.286	
		ADR	T = 0.800	TT = 0.600	
			G = 0.200	TG = 0.400	
	rs2235048	CTR	C = 0.429	CC = 0.143	C = 0.398/867	Intron between 25 and 26 exon		[C] ↔ [T], 0.682	[CC] ↔ [CT], 0.417	[CC] ↔ [TT], 0.417
			T = 0.571	CT = 0.571	
				TT = 0.286	
		ADR	C = 0.525	CC = 0.300	
			T = 0.472	CT = 0.450	
				TT = 0.250	
*ABCC2*	66744 T > G	CTR	T = 1.000	T/T = 1.000	No reported	1323; T > G; Ile > Ser	Cytoplasmatic region, fourth domain	**[T]** ***↔*** **[G], 4.038**	[TT] ↔ [TG], 4.1714	[TT] ↔ [GG], 0.429
		ADR	T = 0.890	T/T = 0.770	
			G = 0.110	T/A = 0.230	
	rs3740066	CTR	C = 0.928	C/C = 0.857	T = 0.304/663	1324; C > T; Ile > Ile	Cytoplasmatic region, fourth domain	**[C]** ***↔*** **[T], 4.875**	[CC] ↔ [CT], 2.769	[CC] ↔ [TT], 3.370
			T = 0.072	C/T = 0.143	
		ADR	C = 0.727	C/C = 0.591	
			T = 0.273	C/T = 0.273	
				T/T = 0.136	
	68049 T > A	CTR	T = 0.790	TT = 0.570	No reported	1373; T > A; Leu > His	Cytoplasmatic region, fourth domain	[T] ↔ [A], 0.733	[TT] ↔ [TA], 0.667	[TT] ↔ [AA], 0.310
			A = 0.210	TA = 0.430	
		ADR	T = 0.840	TT = 0.680	
			A = 0.160	TA = 0.320	
	67967C > A	CTR	C = 0.500	CA = 1.000	No reported	1342; C > A; Ser > Ser	Cytoplasmatic region, fourth domain	[C] ↔ [A], 0.760	[CC] ↔ [CA], 0.371	[CC] ↔ [AA], 0.143
			A = 0.500	
		ADR	C = 0.570	CC = 0.140	
			A = 0.430	CA = 0.860	
	68072C > A	CTR	C = 0.500	CA = 1.000	No reported	1381; C > A; Pro > Thr	Cytoplasmatic region, fourth domain	[C] ↔ [A], 0.750	[CC] ↔ [CA], 0.352	[CC] ↔ [AA], 0.143
			A = 0.500	
		ADR	C = 0.590	CC = 0.180	
			A = 0.410	CA = 0.820	
	68088 G > C	CTR	G = 0.710	GG = 0.710	No reported	Intron between 29 and 30		[G] ↔ [C], 0.147	[GG] ↔ [GC], 0.314	[GG] ↔ [CC], 0.147
			C = 0.290	CC = 0.290	
		ADR	G = 0.890	GG = 0.840	
			C = 0.110	CC = 0.160	

*Relative frequencies are presented for the five SNPs reported on NCBI Gene Bank database of ABCB1 and ABCC2 genes among 14 controlled and 44 drug-resistant patients (2n), obtained by direct sequencing. The position indicates the nucleotide change at ABCC2 gene from RefSeqGene NG_011798.1. Amino acid change was obtained from SNP page and its localization from http://www.uniprot.org/ website. Odds ratio (OR) represent the allele risk or mutated using the software on line through the web page http://ihg.gsf.de/cgi-bin/hw/hwa1.pl. Bold font: allele OR > 1.0. DR, drug-resistant; CTR, controlled; MAF, Minor Allele Frequency*.

Another comparison was made between the relative frequencies of the minor allele (MAF) in our population and in the population represented by the NCBI GenBank data. The most significant similarities were found in rs1128503 and rs3740066 in ADR and in rs1045642 and rs2235048 in CTR (Table 3).

An important aspect of genetic alteration is whether a nucleotide change results in a change in an amino acid residue and whether this, in turn, structurally modifies the protein. In this respect, the SNPs rs2229109 and rs2032582 are of interest due to the possible modifications that they may produce in protein MDR1 (Table 3).

The relative frequencies of the studied alleles were also compared with their frequencies in the Hispanic and Mexican reference populations reported in the NCBI GenBank (Table 4). In our population, SNP rs2229109 in exon 10 of *ABCB1* occurred with a relative frequency of 0.09 and was only present in heterozygous form in the ADR group (Table 3). Although a reference for the Mexican population was not available, we observed that our results showed a twofold higher minor allele frequency with respect to sample ss48292345 from the Hispanic population (0.043) in a similar number of individuals (Table 4). The SNPs rs1128503 and rs2032582 in exons 11 and 20, respectively, of the *ABCB1* gene showed similar relative frequencies in the CTR (0.71) and ADR (0.82) patient groups; however, there was a difference in the frequencies of homozygotes and heterozygotes in the CTR group (Table 3). Furthermore, there were no Mexican individuals with AG heterozygous mutations in SNP rs2032582, and the AT heterozygous mutation was absent from both populations (Table 4).

**Table 4:** **Genotype and allelic population frequencies of SNPs found in ABCB1 and ABCC2 genes**.

Gene	SNP	NCBI assay ID	Population	Sample (2n)	Allele frequency	Genotype frequency
ABCB1	rs2235048	ss35072278	Hispanic	44	T = 0.455	TT = 0.182
					C = 0.545	TC = 0.545
						CC = 0.273
		ss48292340	Hispanic	38	T = 0.553	TT = 0.369
					C = 0.447	TC = 0.368
						CC = 0.263
		ss44833502	Mexican	100	T = 0.540	TT = 0.260
					C = 0.460	TC = 0.560
						CC = 0.180
	rs2235047	ss35072277	Hispanic	44	T = 0.932	TT = 0.864
					G = 0.068	TG = 0.136
			Mexican	100	T = 0.900	TT = 0.840
					G = 0.100	TG = 0.126
						GG = 0.011
		ss48292339	Hispanic	42	T = 0.952	TT = 0.905
					G = 0.048	TG = 0.095
						GG = 0.00
	rs1045642	ss35072275	Hispanic	42	T = 0.452	TT = 0.238
					C = 0.548	TC = 0.429
						CC = 0.333
			Mexican	100	C = 0.540	TT = 0.180
					T = 0.460	TC = 0.560
						CC = 0.260
	rs2214102	ss12675208	Hispanic	46	G = 0.957	G/G = 0.913
					A = 0.043	A/G = 0.087
		ss35071953	Hispanic	44	G = 0.905	G/G = 0.810
					A = 0.095	A/G = 0.190
		ss52073858	Mexican	77	G = 0.980	G/G = 0.960
					A = 0.020	A/G = 0.040
	rs2229109	ss35072104	Hispanic	44	G = 1.000	G/G = 1.000
					A = 0.000	A/G = 0.000
		ss48292345	Hispanic	46	G = 0.978	G/G = 0.957
					A = 0.022	A/G = 0.043
	rs1128503	ss35072107	Hispanic	44	T = 0.545	C/T = 0.545
					C = 0.455	T/T = 0.273
						C/C = 0.182
		ss48292343	Hispanic	46	C = 0.609	C/T = 0.522
					T = 0.391	C/C = 0.348
						T/T = 0.130
		ss52068966	Mexican	77	C = 0.540	C/T = 0.560
					T = 0.460	C/C = 0.260
						T/T = 0.180
	rs2032582	ss12675210	Hispanic	46	G = 0.565	G/T = 0.522
					T = 0.392	G/G = 0.261
					A = 0.043	T/T = 0.130
						A/G = 0.08
		ss35072218	Hispanic	44	G = 0.545	G/T = 0.636
					T = 0.455	G/G = 0.227
						T/T = 0.136
		ss12675210	Mexican	77	G = 0.570	G/T = 0.540
					T = 0.430	G/G = 0.300
						T/T = 0.160
ABCC2	rs3740066	ss48292395	M	44	C = 0.614	C/T = 0.500
					T = 0.386	C/C = 0.364
						T/T = 0.136

*Hispanic populations from ss12675208, ss48292345, ss48292343, ss12675210, and ss48292395 samples belong to HISP-MULTI-NATIONAL Population ID Class, obtained of 23 anonymized individuals of self-described HISPANIC heritage. Hispanic populations from ss35071953, ss35072104, ss35072107 and ss35072218 samples belong to EGP_HISP-PANEL Population ID Class, obtained of 22 individuals with Coriell Cell Repository (CCR) ID. Mexican population belongs to HAPMAP-MEX Population ID Class, obtained of 100 individuals with Mexican ancestry in Los Angeles, California*.

In studies of patients with ADR, SNP rs1045642 in exon 25 has been shown to be relevant in association studies with drug resistant; in our population, the relative frequencies of this SNP were similar in the CTR and ARE groups at both the allelic and the genotypic levels. Furthermore, the SNPs rs2235047 and rs2235048 were identified in an intron between exons 25 and 26. The heterozygous form of rs2235047 showed a higher frequency ADR than in CTR (0.400 vs. 0.286), as seen in Table 3; however, the most outstanding difference in this SNP was in comparison to reference populations, relative to which it was more than three times as frequent (Table 4); this is more significant in our population than rs1045642. The relative frequency of SNP rs2235048 was the same as that of rs1045642; when SNP rs1045642 showed the TT, TC, or CC genotype, we found the genotype CC, CT, or TT, respectively, in the position of SNP rs2235048. This association may be relevant to our population, which so far has not shown a clear similarity to reference populations.

SNP rs3740066 in exon 28 of the *ABCC2* gene occurred with a relative frequency of 0.13 as a heterozygous polymorphism in the controlled group and with a relative frequency of 0.41 as homozygous and heterozygous polymorphisms in the drug-resistant group; the observed heterozygous ratio (ADR: CTR) was 2:1. In addition, the unexpected SNP rs2214102 located in the 5′UTR region of the *ABCB1* gene was present in the homozygous form in both groups; only 5% of the drug-resistant patients had the heterozygous form. However, this finding is not relevant because it is similar to a previous report in a Mexican population.

Some other differences observed in this study are worth noting. At SNP rs2032582, the allelic and genotypic distribution in the controlled group was more similar to reported populations in NCBI GenBank than was that of the ADR; in particular, the heterozygous A > T mutation was not found in other populations similar to ours. Furthermore, although allele A of SNP rs2229109 was present at a lower frequency, our population showed an allele frequency more than 2.5-fold greater than the reported MAF (0.045 vs. 0.017).

### New polymorphisms of the *ABCC2* gene in Mexican epileptic pediatric patients

**Figure 1:**
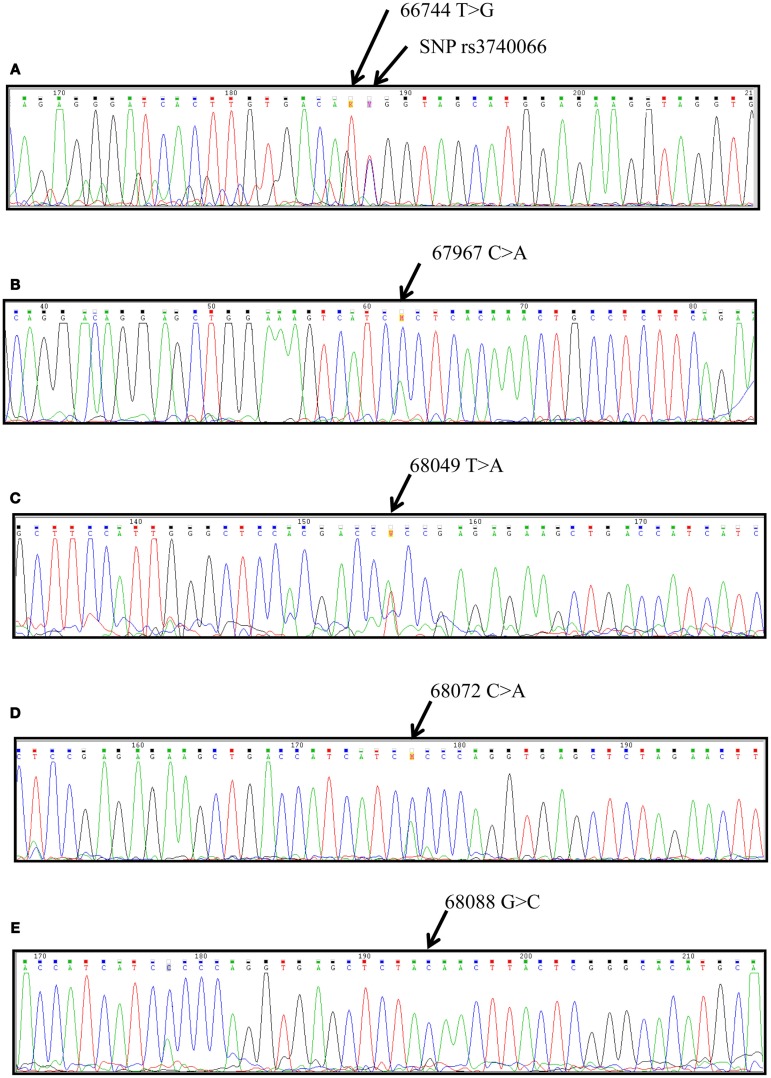
**Chromatograms of single-nucleotide changes found in the *ABCC2* gene from Mexican pediatric patients with epilepsy**. **(A)** The 66744 T > G change in located in exon 28. **(B)** The 67967 C > A change in located in exon 29. **(C)** The 68049 T > A change in located in exon 29. **(D)** The 68072 C > A change in located in exon 29. **(E)** The 68088 G > C change in located in intron 29. The position indicates the nucleotide change in the ABCC2 gene (RefSeqGene NG_011798.1).

By sequencing amplicons from exons 28 and 29 of the *ABCB1* and *ABCC2* genes, we found other single-nucleotide changes (SNCs) that have not been reported in the NCBI GenBank database (Table 2, RefSeqGene NG_011798.1). The SNC located in position 66744T > G adjacent to rs3740066 in exon 28 is present as a homozygous wild-type or heterozygous mutation, allowing the formation of four different triplets: ATC, ATT, AGT, and AGC. The ATC and ATT triplets, which show relative frequencies of 0.929 and 0.682 for triplet ATC and 0.071 and 0.205 for triplet ATT in CTR and ADR, respectively, are not associated with an amino acid change (Ile1324Ile). The AGT and AGC triplets produce an amino acid change (Ile1324Ser, Figure 1A); these occur at relative frequencies of 0.068 and 0.045, respectively, but only in drug-resistant patients.

For the SNC 67967C > A in exon 29, no amino acid change (Ser1342Ser) was observed due to nucleotide substitution (Figure 1B); however, SNC 68072C > A resulted in the amino acid change Pro1381Thr (Figure 1D). This allele was present as a heterozygous mutation in both groups (1.0 in the CTR group, 0.86 in the ADR group). The SNC 68049T > A in exon 29 resulted in a Leu1373His change (Figure 1C); this allele was heterozygous (0.43 in the CTR group, 0.32 in the ADR group), but the wild-type allele predominated in both groups (>50%). These nucleotide changes are located in the region of the gene coding for the fourth domain of the protein, corresponding to its cytoplasmic region. In contrast, the SNC 68088G > C (Figure 1E), which is located between exons 29 and 30, occurred in our study population as a homozygous mutation with a relative frequency of 0.29 in the CTR group and a relative frequency of 0.05 in ADR group.

## Discussion

The pediatric epilepsy patients admitted to the Neurology Service, Hospital of Pediatrics, National Medical Center, Century XXI, were referred from family clinics and general hospitals for inadequate control of epileptic seizures. In most cases, the patients received dosage adjustments or changes in drug regimen, drug monitoring, and electroencephalographic studies and were followed closely for adequate control of their seizures. However, some patients continued to interictal epileptiform discharges on EGG and/or the occurrence of seizures, and some gradually deteriorated. The results obtained with a small group of patients who did not respond to at least three different drug regimens but were followed for 4.7 ± 3.8 (CI = 1–14) years are presented in this work.

Fagiolino et al. (23) suggested that the quantitation of drugs in saliva can be used to identify drug-resistant patients. The hypothesis presented by these authors is based on the drug concentration circulating in free plasma, which irrigates the glandular capillary tissue, resulting in equilibrium with the aqueous spaces. Considering our results with respect to this hypothesis, we found a lower concentration of free VPA in drug-resistant patients, suggesting a possible deficiency in drug transport in these patients.

The overexpression of P-gp and MRP2 in resected tissue from patients who have undergone surgery for epilepsy suggests that this phenomenon may be conditional upon the presence of polymorphisms in the genes *ABCB1* and *ABCC2* and not simply to exposure to xenobiotics that recognize these transporter proteins. However, the association between polymorphisms of the *ABCB1* and *ABCC2* genes and anti-epileptic drug-resistant epilepsy is controversial; SNPs rs1128503 (C1236T), rs2032582 (G2677T/A), and especially rs1045642 (C3435T) in the *ABCB1* gene have not shown a clear relationship to anti-epileptic drug-resistant epilepsy from different populations or ethnic groups (13, 24–27). This may be because, as has been reported, SNP rs1045642 is more relevant as a haplotype with rs1128503 and rs2032582. Zimprichet et al. (28), studying European patients with temporal lobe epilepsy, demonstrated a relationship between the CGC haplotype at SNPs rs1128503, rs2032582, and rs1045642, respectively, and resistance to several AEDs. In another report, Seo et al. (10) demonstrated that Japanese with anti-epileptic drug-resistant epilepsy showed a tendency to possess the haplotype TTT at SNPs rs1128503, rs2032582, and rs1045642 when CBZ was employed. To enable comparison with those reports, haplotype frequency was calculated in our population. Both haplotypes (CGC and TTT) were found in our population. Within each group, the two haplotypes occurred with similar frequencies (CGC = 0.619, TTT = 0.523 in ADR and CGC = 0.714, TTT = 0.714 in CTR), but both were present at higher frequencies in the CTR group.

For patients who exhibit a mutated allele, OR values can be used to represent relative risk factors among the ADR and CTR patients. The polymorphisms or nucleotide changes, in descending order of risk factor magnitude, are rs2214102 > rs2032582 [T ↔ A] > rs2229109 > rs1128503 > rs2032582 [T ↔ G] > rs2235047 in *ABCB1*, and rs3740066 > 66744 T > G in *ABCC2*; relative to heterozygote genotype rs2032582 [T ↔ A] > rs2214102 > rs2229109 > rs2032582 [T ↔ G] > rs11 28503 > rs2235047 in *ABCB1*, and 66744 T > G > rs3740066 in *ABCC2*; finally, with regard to the homozygote genotype, rs1128503 > rs2032582 [T ↔ G] in *ABCB1* and rs3740066 in *ABCC2* (Table 3).

Although the number of patients is lower in our work, the results are similar to studies with larger numbers of patients (25, 29, 30), where the lack of association of *ABCB1* polymorphisms with anti-epileptic drug resistance (indicated by us as a risk factor) it has been pointed out, including two Indian studies (31, 32) and a meta-analysis (26). In contrast, Taur et al. (33) and Siddiqui et al. (34) reported association with anti-epileptic drug resistance. On the other hand, we find that the *ABCC2* is a greater risk factor for increased anti-epileptic drug resistance than *ABCB1*, although in previous studies, we only share the SNP rs3740066, not representing a major risk factor as the high activity promoter variant c-24C > T; these data suggest that *ABCC2* polymorphism may affect the response to AEDs.

This study employed a different strategy, rigorously selecting pediatric patients with ADR and patients with good seizure control to decrease the potential influence of confounding variables associated with the disease, to develop a study utilizing a non-inferential exploratory search based on the polymorphisms already reported and on unreported new characteristics of the Mexican population. Because our population has been little studied, this will allow us to conduct association studies with new SNPs and AEDs used to treat different forms of epilepsy. To avoid sequencing entire genes, we selected exons containing polymorphisms that have been previously reported in Mexican and Hispanic populations, including the P-gp and MRP2 proteins, which are associated with drug resistance. The most important outcome of this study was the identification of five new changes in the *ABCC2*; these are 66744T > G, 67967C > A, 68072C > A, 68049C > A, and G68088C. The nucleotide change 66744T > G was found only in ADR, and the risk factor associated with it was one of the highest found in the study. Moreover, the genetic variability in SNP rs2032582 in ADR was different and unexpected based on previous reports in the Mexican population in that the A allele was found. This result requires replication in a larger pool of Mexican patients.

## Conflict of Interest Statement

The authors declare that the research was conducted in the absence of any commercial or financial relationships that could be construed as a potential conflict of interest.

## References

[1] Ngugi AK Bottomley C Kleinschmidt I Sander JW Newton CR Estimation of the burden of active and life-time epilepsy: a meta-analytic approach. Epilepsia (2010) 51:883–90 10.1111/j.1528-1167.2009.02481.x 20067507 PMC3410521

[2] World Health Organization (WHO). Epilepsy: Fact Sheet No 999. (2012). Available from: http://www.who.int/mediacentre/factsheets/fs999/en/index.html

[3] Kwan P Baum L Wong V Ng PW Lui CH Sin NC Association between ABCB1 C3435T polymorphism and drug-resistant epilepsy in Han Chinese. Epilepsy Behav (2007) 11:112–7 10.1016/j.yebeh.2007.04.013 17521963

[4] Stepien KM Tomaszewski M Tomaszewska J Czuczwar SJ The multidrug transporter P-glycoprotein in pharmacoresistance to antiepileptic drugs. Pharmacol Rep (2012) 64:1011–9 10.1016/S1734-1140(12)70900-3 23238460

[5] Aller SG Yu J Ward A Weng Y Chittaboina S Zhuo R Structure of P-glycoprotein reveals a molecular basis for poly-specific drug binding. Science (2009) 323:1718–22 10.1126/science.1168750 19325113 PMC2720052

[6] Rivers F O’Brien TJ Callaghan R Exploring the possible interaction between anti-epilepsy drugs and multidrug efflux pumps; in vitro observations. Eur J Pharmacol (2008) 598:1–8 10.1016/j.ejphar.2008.09.014 18835265

[7] Löscher W Klotz U Zimprich F Schmidt D The clinical impact of pharmacogenetics on the treatment of epilepsy. Epilepsia (2009) 50:1–23 10.1111/j.1528-1167.2008.01716.x 18627414

[8] Moerman L Wyffels L Slaets D Raedt R Boon P De Vos F Antiepileptic drugs modulate P-glycoproteins in the brain: a mice study with 11C-desmethylloperamide. Epilepsy Res (2011) 94:18–25 10.1016/j.eplepsyres.2010.12.013 21277169

[9] Aronica E Sisodiya SM Gorter JA Cerebral expression of drug transporters in epilepsy. Adv Drug Deliv Rev (2012) 64:919–29 10.1016/j.addr.2011.11.008 22138133

[10] Seo T Ishitsu T Ueda N Nakada N Yurube K Ueda K ABCB1 polymorphisms influence the response to antiepileptic drugs in Japanese epilepsy patients. Pharmacogenomics (2006) 7:551–61 10.2217/14622416.7.4.551 16753003

[11] Kwan P Arzimanoglou A Berg AT Brodie MJ Allen Hauser W Mathern G Definition of drug resistant epilepsy: consensus proposal by the ad hoc task force of the ILAE commission on therapeutic strategies. Epilepsia (2010) 51:1069–77 10.1111/j.1528-1167.2009.02397.x 19889013

[12] Nurmohamed L Garcia-Bournissen F Buono RJ Shannon MW Finkelstein Y Predisposition to epilepsy does the ABCB1 gene play a role? Epilepsia (2010) 51:1882–5 10.1111/j.1528-1167.2010.02588.x 20491876

[13] Haerian BS Lim KS Mohamed EH Tan HJ Tan CT Raymond AA Lack of association of ABCB1 and PXR polymorphisms with response to treatment in epilepsy. Seizure (2011) 20:387–94 10.1016/j.seizure.2011.01.008 21316268

[14] Haerian BS Lim KS Tan HJ Mohamed EH Tan CT Raymond AA Association between ABCB1 polymorphism and response to sodium valproate treatment in Malaysian epilepsy patients. Epileptic Disord (2011) 13:65–75 10.1684/epd.2011.0419 21388909

[15] Kumari R Lakhan R Garg RK Kalita J Misra UK Mittal B Pharmacogenomic association study on the role of drug metabolizing, drug transporters and drug target gene polymorphisms in drug-resistant epilepsy in a north Indian population. Ind J Hum Genet (2011) 1:S32–40 10.4103/0971-6866.80357 21747585 PMC3125053

[16] Sayyah M Kamgarpour F Maleki M Karimipoor M Gharagozli K Shamshiri AR Association analysis of intractable epilepsy with C3435T and G2677T/A ABCB1 gene polymorphisms in Iranian patients. Epileptic Disord (2011) 13:155–65 10.1684/epd.2011.0443 21636342

[17] Sterjev Z Trencevska GK Cvetkovska E Petrov I Kuzmanovski I Ribarska JT The association of C3435T single-nucleotide polymorphism, Pgp-glycoprotein gene expression levels and carbamazepine maintenance dose in patients with epilepsy. Neuropsychiatr Dis Treat (2012) 8:191–6 10.2147/NDT.S28285 22570551 PMC3346059

[18] Kim WJ Lee JH Yi J Cho YJ Heo K Lee SH A nonsynonymous variation in MRP2/ABCC2 is associated with neurological adverse drug reactions of carbamazepine in patients with epilepsy. Pharmacogenet Genomics (2010) 20:249–56 10.1097/FPC.0b013e328338073a 20216337

[19] Ufer M von Stülpnagel C Muhle H Haenisch S Remmler C Majed A Impact of ABCC2 genotype on antiepileptic drug response in Caucasian patients with childhood epilepsy. Pharmacogenet Genomics (2011) 21:624–30 10.1097/FPC.0b013e3283498131 21799461

[20] Qu J Zhou BT Yin JY Xu XJ Zhao YC Lei GH ABCC2 polymorphisms and haplotype are associated with drug resistance in Chinese epileptic patients. CNS NeurosciTher (2012) 18:647–51 10.1111/j.1755-5949.2012.00336.x 22630058 PMC6493375

[21] Ramos-Lizana J Aguirre-Rodríguez J Aguilera-López P Cassinello-García E Recurrence risk after a first remote symptomatic unprovoked seizure in childhood: a prospective study. Dev Med Child Neurol (2009) 51:68–73 10.1111/j.1469-8749.2008.03124.x 19021679

[22] Martínez-Juárez IE López-Zapata R Gómez-Arias B Bravo-Armenta E Romero-Ocampo L Estévez-Cruz Z Epilepsia farmacorresistente: uso de la nueva definición y factores de riesgo relacionados. Estudio en población mexicana de un centro de tercer nivel. Rev Neurol (2012) 54:159–66 22278892

[23] Fagiolino P Vázquez M Maldonado C Aspectos farmacocinéticos del tratamiento antiepiléptico y su monitoreo mediante el uso de saliva. In: Beas Zárate C Ureña Guerrero M, editors. En: Tópicos de Actualización en Neurobiología: Excitotoxicidad y Cognición en Enfermedades Neurogenerativas: Aspectos Básicos, Clínicos y Sociales. Guadalajara: Universidad de Guadalajara (2010). p. 381–400

[24] Chen P Yan Q Xu H Lu A Zhao P The effects of ABCC2 G1249A polymorphism on the risk of resistance to antiepileptic drugs: a meta-analysis of the literature. Genet Test Mol Biomarkers (2014) 18:106–11 10.1089/gtmb.2013.0362 24325761 PMC3926164

[25] Grover S Bala K Sharma S Gourie-Devi M Baghel R Kaur H Absence of a general association between ABCB1 genetic variants and response to antiepileptic drugs in epilepsy patients. Biochimie (2010) 92:1207–12 10.1016/j.biochi.2010.04.008 20417680

[26] Haerian BS Roslan H Raymond AA Tan CT Lim KS Zulkifli SZ ABCB1 C3435T polymorphism and the risk of resistance to antiepileptic drugs in epilepsy: a systematic review and meta-analysis. Seizure (2010) 19:339–46 10.1016/j.seizure.2010.05.004 20605481

[27] Dong L Luo R Tong Y Cai X Mao M Yu D Lack of association between ABCB1 gene polymorphisms and pharmacoresistant epilepsy: an analysis in a western Chinese pediatric population. Brain Res (2011) 1391:114–24 10.1016/j.brainres.2011.03.028 21420937

[28] Zimprich F Sunder-Plassmann R Stogmann E Gleiss A Dal-Bianco A Zimprich A Association of an ABCB1 gene haplotype with pharmacoresistance in temporal lobe epilepsy. Neurology (2004) 63:1087–9 10.1212/01.WNL.0000141021.42763.F6 15452305

[29] Tan NC Heron SE Scheffer IE Pelekanos JT McMahon JM Vears DF Failure to confirm association of a polymorphism in ABCB1 with multi-drug resistant epilepsy. Neurology (2004) 63:1090–2 10.1212/01.wnl.0000137051.33486.c7 15452306

[30] Szoeke C Sills GJ Kwan P Petrovski S Newton M Hitiris N Multidrug-resistance genotype (ABCB1) and seizure recurrence in newly treated epilepsy: data from International Pharmacogenetic Cohorts. Epilepsia (2009) 50:1689–96 10.1111/j.1528-1167.2009.02059.x 19453704

[31] Lakhan R Misra UK Kalita J Pradhan S Gogtay NJ Singh MK No association of ABCB1 polymorphisms with drug-refractory epilepsy in a north Indian population. Epilepsy Behav (2009) 14:78–82 10.1016/j.yebeh.2008.08.019 18812236

[32] Vahab SA Sen S Ravindran N Mony S Mathew A Vijayan N Analysis of genotype and haplotype effects of ABCB1 (MDR1) polymorphisms in the risk of medically refractory epilepsy in an Indian population. Drug Metab Pharmacokinet (2009) 24:255–60 10.2133/dmpk.24.255 19571437

[33] Taur SR Kulkarni NB Gandhe PP Thelma BK Ravat SH Gogtay N Association of polymorphisms of *CYP2C9, CYP2C19*, and ABCB1, and activity of P-glycoprotein with response to anti-epileptic drugs. J Postgrad Med (2014) 60(3):265–9 10.4103/0022-3859.138739 25121365

[34] Siddiqui A Kerb R Weale ME Brinkmann U Smith A Goldstein DB Association of multidrug resis*tance* in *epilep*sy with a polymorphism in the drug-transporter gene ABCB1. N Engl J Med (2003) 348:1442–8 10.1056/NEJMoa021986 12686700

